# Statistical analysis plan for the FiO_2_-C trial: effects of closed-loop automatic control of the inspiratory fraction of oxygen (FiO_2_-C) on outcomes of extremely preterm infants—a randomized-controlled parallel group multicentre trial for safety and efficacy

**DOI:** 10.1186/s13063-024-08615-7

**Published:** 2024-11-12

**Authors:** Jochem König, Anette Stauch, Corinna Engel, Michael S. Urschitz, Axel R. Franz, for the FiO2-C study group, Jochem König, Anette Stauch, Corinna Engel, Michael S. Urschitz, Axel R. Franz, Christian F. Poets, Helmut Hummler, Hendrik J. Niemarkt, Dirk Bassler, Christian A. Maiwald, Iris Bergmann, Monika Weiss, Andreas Eichhorn, Michael Raubuch, Michael Roth, Birgit Schuler, Kai Rötsch, Ruimiao Bai, Andreas Fiedler, Sonja Kapp, Thomas M. K. Völkl, Sibylle C. Horsinka, Edmondo N. L. Hammond, Christoph von Buch, Hans Thorsten Körner, Birte Tröger, Mario Rüdiger, Barbara Seipolt, Lars Mense, Thomas Hoehn, Klaus Lohmeier, Hans-Jörg Bittrich, Kathrin Roefke, Klaus Niethammer, Britta Brenner, Olaf Raecke, Hans Fuchs, Daniel Klotz, Anna Koluch, Sandra Idel, Laura Lübking, Bettina Bohnhorst, Corinna Peter, Christoph Jacobi, Christian Gille, Bernd Beedgen, Tina Heinzmann, Sascha Meyer, Joachim Kühr, Sandra Holz, Silvia Welcker, Ulrich H. Thome, Benjamin W. Ackermann, Corinna Gebauer, Andreas W. Flemmer, Susanne Herber-Jonat, Adelheid Kley, Marcus Krüger, Daniela Reber, Marcus Krüger, Christian Brickmann, Kilian Ackermann, Julia Sandkötter, Katja Masjosthusmann, Michael Schroth, Christian Grillhösl, Jochen Kittel, Holger Michel, Hans-Christoph Schneider, Anja Mayer, Hans-Martin Lode, Daniel Lorenz, Axel T. Bosk, Hans-Jürgen Gausepohl, Torben Lindner, Matthias Vochem, Thomas Strahleck, Patrick Neuberger, Christian A. Maiwald, Jörg Arand, Harald Ehrhardt, Marc R. Mendler, Jochen Essers, Christian Bender, Jessica Beckmann, Narmina Mammodova, Ralf Rauch, Ulrich Bernbeck, Hendrik J. Niemarkt, Thilo Mohns, Estelle E. M. Mulder, Henrica L. M. van Straaten, Matthias Hütten, Elke van Westering-Kroon, Vrinda Nair

**Affiliations:** 1 Division of Paediatric Epidemiology, Institute of Medical Biostatistics, Epidemiology, and Informatics, University Medical Centre of the Johannes Gutenberg University Mainz, Mainz, Germany; 2 Centre for Pediatric Clinical Studies (CPCS), University Children’s Hospital Tübingen, Calwerstr. 7, Tübingen, 72076 Germany; 3 Department of Neonatology, University Children’s Hospital Tübingen, Tübingen, Germany

**Keywords:** Closed-loop automatic control of the inspiratory fraction of oxygen (FiO_2_-C), Infant, Premature, Intermittent hypoxemia and hyperoxemia, Oxygen

## Abstract

**Background:**

Extremely low gestational age neonates (ELGANs, i.e. those born before 28 weeks postmenstrual age (PMA)) often require supplemental oxygen and frequently experience intermittent hypo- and hyperoxemic episodes. Exposure to episodes with inadequate oxygen concentrations has been shown to be associated with an increased risk of retinopathy of prematurity (ROP), bronchopulmonary dysplasia (BPD), necrotizing enterocolitis (NEC), neurodevelopmental impairment (NDI) and death. Closed-loop automatic control of the inspiratory fraction of oxygen (FiO_2_-C) reduces number and duration of hypo- and hyperoxemic episodes in ELGANs. Its impacts on clinically important short- and long-term outcomes such as ROP, BPD, NEC, NDI and mortality have not yet been studied.

**Methods:**

An outcome-assessor-blinded, multicentre, randomized-controlled, parallel-group trial for superiority was designed to study the effects of FiO_2_-C (provided by standard infant ventilators) in addition to routine manual control (RMC) during respiratory support, compared to RMC only, on short- and long-term clinical outcomes in ELGANs. Two co-primary composite outcomes were defined: (i) death, severe ROP, BPD or NEC, assessed at 36 weeks PMA or, in case of ROP, until complete vascularization of the retina; (ii) death or NDI (defined as language/cognitive delay, motor impairment, severe visual impairment or hearing impairment), assessed at 2 years corrected age.

**Results:**

Primary outcomes will be compared between the two intervention groups using a Cochran-Mantel-Haenszel test. The factors considered for randomization (centre, sex and gestational age at birth (< 26 weeks and ≥ 26 weeks)) will be used to define strata. Results will be presented as adjusted odds ratios with two-sided 95% and 97.5% confidence intervals and two-sided *p* values.

**Conclusions:**

The statistical analyses for the FiO_2_-C trial were defined in the study protocol and specified in detail in this statistical analysis plan published prior to any statistical analysis. This is in accordance with the Declaration of Helsinki and the International Conference on Harmonization Good Clinical Practice guidelines.

**Trial registration:**

ClinicalTrials.gov NCT03168516. Registered on May 30, 2017.

## Introduction

### Background and rationale

Approximately 0.5% of all neonates (i.e. about 25,000 infants per year in Europe) are extremely low gestational age neonates (ELGANs), i.e. have a gestational age (GA) of < 28 completed weeks at birth. Mortality effects 10–20% of ELGANs [1–5]. Severe retinopathy of prematurity (ROP > stage 2), chronic lung disease of prematurity (also referred to as bronchopulmonary dysplasia (BPD)) and necrotizing enterocolitis (NEC) > stage 1 affect 8–19%, 38–44% and 8–12% of ELGANs, respectively [1, 6, 7]. Severe neurodevelopmental impairment (NDI) affects about 25–35% of ELGANs [1, 8].

The vast majority of ELGANs require supplemental oxygen in addition to mechanical respiratory support, including invasive and non-invasive ventilation as well as continuous positive airway pressure. In a world-wide collaboration, a higher target range of arterial oxygen saturation by pulse oximetry (SpO_2_) of 91–95% was compared with a lower level (85–89%), and results suggested that the higher target range was associated with reduced mortality, potentially also reduced incidence of NEC, but increased risk of severe ROP and potentially also BPD [1, 6, 7, 9].

The risk of ROP is increased in the presence of recurrent intermittent hypoxemia leading to wide fluctuations in oxygen levels [10–13].

Similarly, several observational studies reviewed in [14] as well as SpO_2_ data from the Canadian Oxygen Trial (COT [1]) further suggest that late deaths (i.e. deaths beyond 36 weeks postmenstrual age (PMA)) and neurodevelopmental impairment (both, cognitive and in particular motor impairment) are also linked to hypoxemic episodes [13], particularly those with more than 60 s duration.

Keeping oxygen levels (usually measured as pulse oximetry-measured oxygen saturation (SpO_2_)) stable despite irregular breathing patterns in ELGANs requires frequent adjustments of the inspiratory fraction of oxygen (FiO_2_), which is challenging, time-consuming and often impossible due to limited personnel resources. Previous studies suggested that the SpO_2_ of ELGANs may be within desired target ranges for only approximately 30% of the time [15].

The proportion of time within SpO_2_ target range can be significantly improved by application of FiO_2_-C [16–26]. It has repeatedly been shown that, in comparison with routine manual control (RMC) of FiO_2_, FiO_2_-C reduces the burden of hypo- and hyperoxemia in infants while being safe and accurate in short-term clinical evaluation studies [27–32]. The effects of FiO_2_-C on clinically relevant short- and long-term outcomes and the patient safety of a permanent use of FiO_2_-C in routine care have yet to be elucidated.

### Objectives and outcomes

This trial will provide insights into the safety and efficacy of FiO_2_-C provided by standard infant ventilators, started shortly after birth and continuously applied while on respiratory support, in addition to RMC of FiO_2_ compared to RMC of FiO_2_ only. The primary research hypothesis was that the implementation of FiO_2_-C as add-on to routine clinical care reduces the proportion of the short-term composite primary outcome (death, severe ROP, BPD or NEC) until 36th week PMA. The co-primary research hypothesis was that FiO_2_-C also reduces the long-term composite outcome death or severe NDI at 2 years corrected age.

## Essentials of study protocol

### Trial design

This is an outcome-assessor-blinded, randomized-controlled, multicentre parallel-group comparison for superiority (evaluating FiO_2_-C in addition to RMC of FiO_2_ in comparison to RMC of FiO_2_ only) in ELGANs. For a detailed list of recruiting centres, refer to section ‘FiO_2_-C study group’. Essential components of the study protocol have been published previously (Maiwald et al., 2019). In 2021, triggered by the COVID-19 pandemic, an amendment (Protocol V5) introduced the PARCA-R parent questionnaire as a method of systematic assessment of cognitive and language development at 2-year follow-up. In 2023, an amendment (Protocol V6) introduced the following change of the analytical strategy: Based on the power re-assessment, the two hierarchical co-primary outcomes were modified to multiple primary outcomes to be tested using a modified Bonferroni-Holm procedure.

### Trial status

The first patient was enrolled on July 1, 2018. In summer 2023, the Data Monitoring Committee (DMC) recommended stopping the trial early because all efforts to increase recruitment failed and it became apparent that the predefined sample size of 2340 infants could not be achieved with the available resources. Subsequently, recruitment was terminated on October 31, 2023, after 1082 patients have been recruited. Two-year follow-up is ongoing. The COVID-19 pandemic did not affect recruitment, but may have had a negative impact on the completeness of the 2-year follow-up data, as outpatient visits were temporarily prohibited. Intervention, compliance and patient background characteristics did not change during the pandemic.

### Eligibility

#### Inclusion criterion

• GA at birth 23 + 0/7 to 27 + 6/7 weeks (i.e. 23 * 7 + 0 = 161 to 27 * 7 + 6 = 195 days of gestation or 23.0 to 27.9 weeks of gestation)

#### Exclusion criteria

- Decision not to provide full life support/decision for palliative care only before study entry
- Severe congenital abnormalities (particularly those affecting respiratory, cardiovascular or gastrointestinal function or long-term neuro-cognitive development, whereas a patent ductus arteriosus or a PFO/ASDII is not considered a congenital anomaly in preterm infants)
- Postnatal age > 48 h
- Missing parental consent
- Lack of device enabling closed-loop automatic control of FiO_2_ before randomization

### Interventions

#### Experimental intervention

Application of FiO_2_-C (provided by standard infant ventilators) in addition to RMC of FiO_2_ during respiratory support from randomization until preferably 32 weeks PMA.

Detailed guidelines on application of FiO_2_-C were provided by the trial coordinating team in device-specific ‘Study Operating Manuals’ as well as the relevant manufacturer’s user guides. Staff had to be trained by the manufacturer on application of FiO_2_-C according to national Medical Device Legislation.

#### Control intervention

Standard clinical care without using FiO_2_-C, i.e. only RMC of FiO_2_.

#### Both interventions

RMC of FiO_2_ in both treatment groups is provided by staff personnel according to local oxygen therapy guidelines and procedures.

### Intervention allocation and blinding

This study is outcome-assessor-blinded, meaning that the personnel performing the ophthalmological examinations throughout the initial hospitalization as well as the personnel performing the neurocognitive evaluation at 2 years corrected age were blinded to the infants’ treatment group assignment. Blinding of staff and parents was not possible with this type of study intervention.

Study participants were randomly assigned in a 1:1 ratio to FiO_2_-C in addition to RMC of FiO_2_ (test intervention) or RMC of FiO_2_ only (control intervention).

A web-based randomization tool provided by the Interdisciplinary Centre of Clinical Studies at the University Medical Centre of the Johannes Gutenberg University Mainz was used for group assignment. This program enabled bound (into the same treatment group) or free (into different treatment groups) randomization of multiples based on parental choice and took the number of available devices enabling FiO_2_-C at the time of randomization into account.

A minimization algorithm was applied to preferentially aim for an even distribution of treatment assignment within two GA strata (i.e. < 26 weeks and ≥ 26 weeks; 1st priority) and two gender strata (2nd priority) within each centre. A value of 26 weeks was chosen as the cut-off for gestational age at birth, as this approximates the expected median of the sample and is in good agreement with clinical risk stratification and previous large intervention trials in this area.

### Data collection schedule

Data were collected by staff at each participating centre in electronic case record forms (eCRFs) in the study’s secuTrial® electronic database according to the data collection schedule as shown in Table 1.

**Table 1:** Study examinations and data collection

**eCRFs**	**Hospitalization**						**Outpatient**	
	**Birth**		**Study intervention visits**			**Discharge**	**2 years corrected age**	
	**Screening**	**Baseline**	**Visit 3a (d7)**	**Visit 3b (d21)**	**Visit 4 (neonatal outcome)** **PMA 36**	**Discharge (visit 5)**	**Follow-up**	**End of study**
Screening	√							
Randomization	Postnatal age ≤ 48 h							
Baseline		√						
Maternal data		√						
HUS^a^ before randomization		√						
HUS at day 7			√					
HUS at day 21				√				
Neonatal outcome					√			
HUS PMA 36					√			
Oxygen targeting/study intervention		From randomization to 36 weeks PMA						
Concomitant medication		From randomization until discharge						
ROP					≥ PMA 32 until the retina is fully vascularized			
Final ROP assessment					After ROP eCRF has been completed			
Discharge						√		
Follow-up							√	
Bayley^b^							√	
PARCA-R^c^							√	
Incidents and unexpected AEs^d^		From randomization until end of study						
Expected AEs		From randomization until end of study						
End of study								√

^a^ *HUS* head ultrasound, ^b^ *Bayley* Bayley Scales of Infant Development, 3rd edition, ^c^ *PARCA-R* Parent Report of Children’s Abilities-Revised, ^d^ *AE* adverse event

### Interim analysis and stopping rules

No interim analysis has been planned nor conducted for this trial.

### Trial reporting

The trial will be reported according to the CONSORT principles [33]. Final analysis will be done for primary outcome I (and all outcomes secondary to primary outcome I) after database closure for the documentation of in-hospital therapy and outcomes until 36 weeks PMA and for primary outcome II (and its secondary outcomes) after completion of the follow-up of all patients at 2 years corrected age.

## Operational definition of primary and secondary outcomes

### Primary outcomes

Superiority will be assessed for two primary outcomes.

#### Primary outcome I (short-term outcome)

It is a composite of any of the following: (i) death up to 36 weeks PMA, (ii) severe ROP, (iii) BPD or (iv) NEC.

It is coded as ‘present’ (‘1’) if at least one of the four events is documented, as ‘absent’ (‘0’) if all components are documented as ‘absent’, and else as ‘missing/unknown’, coded with ‘9’.

#### Primary outcome II (long-term outcome)

It is a composite of any of the following events: (i) death up to 2 years corrected age or (ii) NDI defined as at least one of the following components at 2 years corrected age:

- Motor disability (defined as a Gross Motor Function Classification System (GMFCS) score of 2–5 [34])
- Language or cognitive delay (language composite score < 85 or cognitive composite score < 85 on Bayley Scales of Infant Development, 3rd edition)
- Severe visual impairment (best corrected vision in the better eye yields a visual acuity less than 6/60 m (20/200 ft) according to the relevant doctor’s reports/discharge summary)
- Hearing impairment (need for a hearing aid or cochlear implant)

It is coded as ‘present’ (‘1’) if at least one of the six events or impairments is documented, as ‘absent’ (‘0’) if all components are documented as absent, and else as ‘missing/unknown’, coded as ‘9’.

If the Bayley III score cannot be used for scoring language or cognitive delay, the Bayley II or another scaled score, the rating of a health care professional, the PARCA-R parent questionnaire or the rating of the parents is used instead. Therefore, the information on physical and mental disability is classified as based on the following cascade:

1. Bayley III score
2. Bayley II score
3. Another score
4. Rating by a health care professional
5. PARCA-R (standardized cognitive and language score)
6. Rating by parents

### Definitions of components of the primary outcome I

#### Severe ROP

ROP was to be diagnosed at routine ophthalmological examinations, beginning at PMA 32 weeks according to international recommendations and local standards. ROP was to be assessed and classified according to standard practice until the retina was fully vascularized [35]. ROP stage 0, 1 or 2 (in zone 2 or 3) were defined as ‘no/non-severe ROP’. ROP stage 3, 4 or 5, or AP-ROP, or any ROP in zone 1, or any treatment for ROP were defined as ‘severe ROP’. Full vascularization may occur as late as 44 weeks PMA—and therefore in some patients after discharge home. Severe ROP for each reported examination is classified as present if scoring indicates it for at least one eye and absent if absence is concluded for both eyes, and missing otherwise.

Documentation of routine eye examinations is defined as conclusive if severe ROP is stated at least once or information is never missing until full vascularization. A variable will be derived that indicates whether data on routine eye examinations were conclusive. If they were not, the conclusion on severe ROP is based on a final ROP assessment from the centre.

#### BPD

According to the physiological definition of Walsh et al. [36], BPD (also termed as moderate or severe BPD in the international classification) is defined as the need for positive pressure support or supplemental oxygen at 36 weeks ± 2 days PMA, including failure in an oxygen reduction test for infants requiring less than 0.3 FiO_2_.

#### NEC

NEC is an important differential diagnosis in premature infants presenting with acute abdomen. The occurrence of NEC, (F)IP or any abdominal surgery will be documented until 36 weeks PMA.

NEC will be defined as modified Bell stage ≥ IIA according to Bell et al. [37] and separated from focal intestinal perforation and other origins of isolated (i.e. non- or pauci-inflammatory) intestinal perforation.

### Secondary outcomes

#### Outcomes secondary to primary outcome I (i.e. components of primary outcome I and closely related variables)

1. Death until PMA 36 weeks
2. ROP severity score (also entitled ‘ROP activity and structure score’, see [38])
3. Severe ROP
4. Treated ROP (laser coagulation, anti-VEGF treatment, cryocoagulation, vitrectomy, cerclage, etc.)
5. BPD
6. NEC

#### Outcomes secondary to primary outcome II (i.e. components of primary outcome II and closely related variables)

7. Death until 2 years corrected age
8. NDI
9. Language composite score < 85
10. Language composite score
11. Cognitive composite score < 85
12. Cognitive composite score
13. Cerebral palsy (CP) as defined by the European network ‘Surveillance of CP in Europe’
14. Motor disability (GMFCS 2–5)
15. GMFCS score
16. Motor composite score < 85
17. Motor composite score
18. Severe visual impairment
19. Severe hearing impairment
20. Non-verbal cognitive standard score by parent questionnaire (PARCA-R) < 85
21. Non-verbal cognitive standard score by parent questionnaire (PARCA-R)
22. Language standard score by parent questionnaire (PARCA-R) < 85
23. Language standard score by parent questionnaire (PARCA-R)

For analysis of the secondary outcomes 9, 11 and 16, the following will apply: The estimated grading by healthcare professionals or parents given in case of missing scores is transformed as follows: ‘normal’ to 100, ‘abnormal’ to 60 (mean between lower limit of standardization (50) and upper limit of clearly abnormal scores (two standard deviations below the reference mean = 70)) and ‘suspect’ to 75.

## Analysis populations

### Post-randomization exclusions

No exclusions were made after randomization, with the exception of one patient who was randomized on the basis of verbal consent, which the parents withdrew shortly afterwards.

### Population definitions

#### Intention-to-treat population

The ITT population consists of all patients included into the study by randomization after parental consent was obtained. Patients for whom parental consent was withdrawn are only included up to this date. The basis for the confirmatory analysis of outcome I is the modified ITT population (mITT-1), i.e. ITT data set with non-missing primary outcome I, and correspondingly mITT-2 for primary outcome II.

#### Per protocol population

The PP population consists of all patients of the ITT population that did not show any major protocol deviation (refer to section ‘Protocol non-compliances’).

## Descriptive analyses

According to GCP, tabulation will be done of all documented data for the ITT data set stratified by treatment group. Tabulation of baseline characteristics by treatment group will be additionally performed for:

- The ITT data set with non-missing primary outcome I
- The ITT data set with non-missing primary outcome II
- The PP data set with non-missing primary outcome I
- The PP data set with non-missing primary outcome II

Numerical items will be summarized as number, number missing, mean, standard deviation, minimum, 25th percentile, median, 75th percentile and maximum, if appropriate. Categorical items will be summarized as numbers and percent.

### Representativeness of trial population and participants

Participant’s flow through each stage of the study will be presented in a CONSORT flow chart. Baseline characteristics of infants and their mothers will be described for the ITT population stratified for treatment group.

### Losses to follow-up

For the short-term primary outcome, which is mainly assessed during hospitalization, we expect only rare cases of consent withdrawal or loss to follow-up. However, for the long-term primary outcome, assessed at 2 years corrected age, we expect a certain proportion of patients to withdraw their informed consent or be lost to follow-up. To avoid losses to follow-up, several other sources for information on neurodevelopmental outcome besides regular follow-up at the study centre and performance of Bayley III examination will be considered (refer to section ‘Operational definition of primary and secondary outcomes’).

### Protocol non-compliances

Regular remote and on-site monitoring according to a predefined monitoring manual ensures high quality of the data. The following protocol non-compliances will be listed in the final report:

#### Major

The following protocol deviations are considered as major:

- Participants randomized in error, i.e. not fulfilling all inclusion or fulfilling an exclusion criterion
- Inclusion into the study more than 96 h after birth
- Application of automatic control of FiO_2_ (FiO_2_-C) for less than 80% of the scheduled intervention time interval
- Any automatic control of FiO_2_ in the control group (defined as > 96 h of automatic control of FiO_2_ in the control group)

#### Minor

Minor protocol deviations will not lead to exclusion from PP population. Descriptive statistics of all protocol deviations will be provided by treatment group.

## Control of type I error and sample size rationale

Analyses of both primary outcome variables based on the modified ITT population will be considered confirmatory. To control for type I error—even if power for analysis of the short-term primary outcome would be insufficient—both outcomes will be assessed independently and corrections for multiple (i.e. twofold) testing will be made according to Bonferroni-Holm. Thus, both outcomes will be tested at the two-sided nominal level of 0.025, and in case that treatment should prove effective with respect to outcome I, the nominal level for outcome II will be relaxed to 0.05.

The analysis of the primary outcome based on the PP population and all analyses of the secondary outcomes are exploratory and will be considered remarkable if *p* < 0.05.

Before starting the study, the required sample size was calculated for the primary research hypothesis that the implementation of FiO_2_-C reduces the proportion of the short-term composite outcome (death, severe ROP, BPD or NEC).

The co-primary research hypothesis was that FiO_2_-C also reduces the long-term composite outcome death or severe NDI.

These hypotheses were planned to be assessed as a priori ordered hypotheses, where the co-primary hypothesis would only be tested in a confirmatory manner if the primary hypothesis had been confirmed. Consequently, no correction for multiple testing was planned initially.

We assumed that:

- The proportion of the short-term primary composite outcome of this study is 50% in the control group.
- FiO_2_-C reduces the burden of severe hypoxemia/hyperoxemia by 25–50% and (based on the assumption that 25–50% of the primary outcome I is associated to recurrent hypo-/hyperoxemia) effects a relative risk reduction (RRR) in this outcome by 12.5%.

In summary, we assumed a reduction in the primary outcome I from 50% (in the control group) to 44% in the intervention (FiO_2_-C) group.

Sample size calculations were based on a chi-square test, assuming a power of 80% and a significance level of 5%. Based on these assumptions, 1110 infants were required in each treatment group (total 2220 infants). Because all components of this primary outcome are determined during the initial hospitalization, the proportion of drop-out before ascertainment of the primary outcome was anticipated to be low (< 5%). Hence, a total of 2340 infants were intended to be enrolled and randomized.

Assuming an incidence of 50% for the co-primary outcome in the control group and an RRR of 25% in the FiO_2_-C group, the proposed sample size had a power > 80% to prove this difference even if up to 20% of randomized infants are lost to follow-up until 2 years corrected age.

### Power re-assessment in preparation of potential early stopping recruitment due to limited resources and insufficient recruitment rate in Q1/2023

In autumn 2023, the anticipated time point for stopping recruitment early due to insufficient recruitment so far, 1000 patients were expected to have been recruited, resulting in a power for the primary outcome I of 38%. The actual RRR would have to be 0.20 (instead of 0.125) in order to still have a power of 80%. For the primary outcome II, a power of 80% could be achieved if this outcome was ascertained in at least 60% of the recruited patients. Considering that the probability of late death or disability in the Canadian Oxygen Trial [1] varied between approximately 30% and 75% depending on the time with SpO_2_ < 80%, a RRR of 0.20 would be possible. And, of course, such a reduction in risk is clinically important, as the components of the primary outcomes are devastating complications (necrotizing enterocolitis, bronchopulmonary dysplasia, retinopathy of prematurity or death) and (associated with) long-lasting impairments (cognitive impairment/motor impairment/blindness).

## Comparative analyses

### Primary analysis

####  Primary outcome I + II

Primary outcome I + II will be compared between the two intervention groups using a Cochran-Mantel-Haenszel test. The factors considered for randomization (centre (grouping of centres that use the same ventilator type in the majority of patients randomized to closed-loop automatic control and have randomized less than 20 patients in total), sex and GA at birth (< 26 weeks and ≥ 26 weeks)) will be used to define strata. Results will be reported as adjusted odds ratio with two-sided 95% and 97.5% confidence intervals and two-sided *p* value.

For primary outcome II, treatment groups will be also compared with respect to the quality of information on physical and mental impairment.

#### Secondary outcomes

Proportions of the binary secondary outcomes 1, 3, 4, 5, 6, 7, 8, 9, 11, 13, 14, 16, 18, 19, 20 and 22 (refer to section ‘Operational definition of primary and secondary outcomes’) will be analysed by Cochran-Mantel-Haenszel test.

The other secondary outcomes will be analysed by means of stratified rank test, the van Elteren test [39], with the same stratification factors as used for the corresponding primary outcome.

The GMFCS has to be handled differently due to rarity of higher response categories. The values 2–5 are grouped as one class, which results in values 0–1–2. An unstratified chi-square test for trend will be applied primarily; an ordinal logistic model with factors treatment group, centre, sex and GA will be fitted additionally.

### Secondary analyses

####  Primary outcome I + II

A marginal logistic model will be fitted with primary outcome I or II as dependent variable, respectively, and centre (grouping small centres as described above), GA at birth, sex and treatment group as explanatory variables, where family-ID will be used to define groups with correlated outcome. Marginal effect measures based on the average risk in each treatment group (odds ratio, relative risk, risk difference and number needed to treat with 95% and 97.5% confidence intervals) will be calculated by the delta method as described in Bender and Vervölgyi [40] using the SAS procedure *glimmix* with the ‘random_residual’ statement and the first order residual robust method for standard errors to specify the marginal logistic model.

In further exploratory analyses, we aim to estimate the complier average causal effect using centre, GA at birth and sex as covariables and varying the definition of compliance. For primary outcome II, a further marginal logistic model is fitted, restricted to survivors up to PMA36.

#### Secondary outcomes

The binary secondary outcomes will be analysed by fitting a marginal logistic model with the same specifications as used for the secondary analysis of the corresponding primary outcome I or II. As a secondary analysis for the scoring outcomes, a linear mixed model will be fitted with sex, GA at birth and grouped centre as factor and a random intercept at family level. In order to cope with possible ceiling effects, separate covariance parameters (residual variance and random intercept variance) will be allowed for by treatment group. Effects will be reported as adjusted mean difference and 95% CI.

The secondary outcomes 7–23 will be analysed without statistical (multiple) imputation of missing outcomes. In particular, children who died before follow-up at 2 years corrected age will not be included. The intended interpretation will be primarily to assess the effect of study intervention in survivors. Fatal events later on will be competing risks to the considered outcome and could be considered as extra category. This is not planned because they are expected to be rare and hence do not markedly change results as compared to precluding them from analysis.

All treatment group comparisons with respect to secondary outcomes will be performed without control of multiple type I error and will be reported with two-sided *p* values and 95% confidence intervals without corrections for multiple testing.

### Multivariable and subgroup analyses

Analyses will be performed by generalized logits models according to Bishop et al. [41], with SAS procedure *catmod* or *gee*, adjusted for treatment group.

Relevant factors for multivariable and subgroup analyses are:

1. Sex (male/female)
2. GA at birth (either as < / ≥ 26 weeks or as continuous variable)
3. Presence of severe intraventricular haemorrhage (IVH) (> 2°/ ≤ 2° or no IVH) at baseline (if the baseline head ultrasound is missing, the results of the next head ultrasound (day 7 ± 2) are used)
4. Ventilator type predominantly used

Subgroup analyses will be performed by including appropriate interaction terms. All multivariable analyses will be performed without control of multiple type I error and will be reported with two-sided *p* values and 95% confidence intervals without corrections for multiple testing.

### Missing data and sensitivity analysis

####  Primary outcome I + II

Using the SAS procedure *mi*, a logistic mixed model with the same variables as used for the secondary analysis will be fitted, that contains a random intercept at family level.

For assessing potential bias due to missing outcome, four scenarios with single imputation of missing primary outcome I + II will be considered:

1. Replace missing values by 1 everywhere
2. Replace missing values by 0 throughout
3. Replace missing values by 0 in intervention and by 1 in controls
4. Replace missing values by 1 in intervention and by 0 in controls

Furthermore, multiple imputation will be performed to generate 50 completed ITT data sets with imputed primary outcome I + II. The multiple imputation will be performed with the method of full conditionals, where a logistic model will be specified for each component of the composite outcome with all other components, the treatment group, GA at birth, sex and centre variable will be specified as predictors. (Death until PMA 36 weeks will presumably be complete and will then not be imputed.)

To investigate potential bias due to a developmental assessment not based on Bayley III scores, a primary outcome IIb is calculated in which neurocognitive and motor development is assessed using Bayley III scores only; four scenarios with single imputation of missing primary outcome IIb will be considered:

1. Replace missing values by 1 everywhere
2. Replace missing values by 0 throughout
3. Replace missing values by 0 in intervention and by 1 in controls
4. Replace missing values by 1 in intervention and by 0 in controls

### Safety

The following safety parameters were reported to the DMC after predefined numbers of patients reached 44 weeks PMA:

1. Early deaths (before 44 weeks PMA), late deaths (after 44 weeks PMA until follow-up at 2 years corrected age) and all deaths (from birth to follow-up at 2 years corrected age)
2. Severe ROP
3. BPD
4. NEC
5. Focal intestinal perforation and other origins of isolated (i.e. non- or pauci-inflammatory) intestinal perforation (FIP/IP) requiring laparotomy
6. Patent ductus arteriosus (PDA) requiring treatment occurring between randomization and PMA 36 weeks
7. Severe IVH of grade 3 or 4 according to Papile
8. Cystic periventricular leukomalacia (PVL)
9. Discharge on home oxygen or home positive pressure respiratory support
10. Proportion of patients in healthy status at 44 weeks PMA versus any of the above complication(s) 2–9 or death
11. Proportion of all incidents, serious incidents as well as adverse events (AEs) and serious adverse events (SAEs)

As safety parameters 1 to 4 are also components of the primary outcome I, they will not be reported again as safety parameters. However, safety parameters 5 to 9 and 11 are reported and safety parameter 10 is modified to:

Incidence of healthy status at 36 weeks PMA versus any of the above complication(s) 2–9 or death up to PMA 36 weeks

For binary items recorded for each patient, OR (95% CI) are reported together with absolute and relative frequencies. Proportions of all incidents, serious incidents as well as adverse events (AEs) and serious adverse events (SAEs) are reported in line listings and aggregated summary tabulation.

## Statistical software employed

SAS 9.4 and R Statistical Software (v4.4; R Core Team 2024) will be used for all analyses.

## Additional exploratory analysis

To analyse a possible bias caused by either ‘*suboptimal*’ routine care or ‘*optimized*’ routine care during the study as compared to current standard routine care outside the study, the following analyses will be displayed within the final analyses.

The predicted model-based proportion of patients with primary outcomes within the control group and the interventional group will be calculated for each centre and for groups of centres that are defined by country, recruitment rates, FiO_2_-device and by indicators of quality of routine care using the *labbe* function of the R package meta.

Heterogeneity of treatment effects across centres and countries will be assessed by statistical methods of meta-analysis. The influence of study centre characteristics that indicate routine performance quality will be investigated by means of meta regression methods. Particularly, methods of bivariate meta-analysis will be applied in order to explore the role of performance under control condition as a moderator of the treatment effect. Results are displayed by means of the L’Abbé plot [42].

## Discussion

This is the statistical analysis plan of a pragmatic, multicentre, multinational randomized parallel group clinical trial for superiority designed to assess safety and efficacy of a new technological intervention in neonatal respiratory care at a time where it has not yet become routine care. This manuscript on the SAP and even more the underlying SAP finalized on May 14, 2024, define in detail the execution of the statistical analyses that were outlined in the study protocol. The publication is timely before database closure and start of any outcome analyses.

### Strengths

Both, assessment of safety and efficacy are of importance, before introducing new technology into routine care and before staff loses equipoise due to, e.g. the reduction of workload for manual FiO_2_ adjustments brought along by the technology. Thereby, the conduct of the trial was timely in a unique window of opportunity.

Observer of key outcomes was blinded to group assignment where possible, but investigators, clinical caretakers and parents could not be blinded due to the nature of the intervention. Central, web-based randomization ensured adequate allocation concealment. GCP monitoring and source data verification have been implemented for key components of the trial to ensure high-quality data. The multicentre multinational design strengthens generalizability of results.

Intense collaboration of and communication between trial sites during the trial enabled identification of technical problems with the new technology including work-arounds, notification of manufacturers and authorities about such problems.

### Limitations

The major limitation of this trial is that the pre-defined sample size could not be reached due to still limited availability of the technology in neonatal intensive care units that expressed their interest to participate in the trial before starting. It was attempted to compensate for this problem by recruiting additional trial sites but the necessary recruitment rate could not be reached in a reasonable time period despite all efforts. Nevertheless, the more than 1000 ELGANs recruited and the rigorous design of the trial will enable at least an unbiased estimate of the potential effect size of the intervention.

Another limitation may be that the set-up (including SpO_2_ monitoring, ventilator and FiO_2_-C algorithm, NICU design and staff availability) differed between study sites where details such as discrepancy between multiple SpO_2_ measurements, delays in the closed-loop circuit (transfer of SpO_2_ data to the algorithm, time from increasing FiO_2_ in the ventilator to increased FiO_2_ at the patient, etc.) may impact on safety and efficacy [43, 44]. On the other hand, the trial was designed to assess safety and efficacy of the generic technology in routine use, and multicentricity will improve generalizability.

## Conclusions

All statistical analyses for the FiO_2_-C trial were defined in the published study protocol and described in detail in this statistical analysis plan published prior to any statistical analysis. This is in accordance with the Declaration of Helsinki and the International Conference on Harmonization Good Clinical Practice guidelines.

## Data Availability

Data sharing is not applicable to this article as no datasets were generated or analysed for the current manuscript.

## Abbreviations

AE: Adverse events
AP-ROP: Aggressive posterior retinopathy of prematurity
BPD: Chronic lung disease of prematurity (according to the physiological definition also termed moderate or severe bronchopulmonary dysplasia)
COT: Canadian Oxygen Trial
CP: Cerebral palsy
CPCS: Centre for Pediatric Clinical Studies
DMC: Data Monitoring Committee
eCRF: Electronic case report form
ELGAN: Extremely low gestational age neonate
ET tube: Endotracheal tube
FiO_2_: Fraction of inspired oxygen
FiO_2_-C: Closed-loop automatic control of the inspiratory fraction of oxygen
FIP: Focal intestinal perforation
GA: Gestational age
GMFCS: Gross Motor Function Classification System
HUS: Head ultrasound
ITT: Intention-to-treat
IVH: Intraventricular haemorrhage
mITT: Modified ITT population
NEC: Necrotizing enterocolitis
NDI: Neurodevelopmental impairment
PARCA-R: Parent Report of Children’s Abilities-Revised
PDA: Patent ductus arteriosus
PMA: Postmenstrual age
PP: Per protocol
PVL: Periventricular leukomalacia
RMC: Routine manual control
ROP: Retinopathy of prematurity
RRR: Relative risk reduction
SAE: Serious adverse event
SpO_2_: Peripheral oxygen saturation

## References

[1] Schmidt B, Whyte RK, Asztalos EV, Moddemann D, Poets C, Rabi Y, et al. Effects of targeting higher vs lower arterial oxygen saturations on death or disability in extremely preterm infants: a randomized clinical trial. JAMA. 2013;309(20):2111–20. 23644995 10.1001/jama.2013.5555

[2] Franz AR, Engel C, Bassler D, Rudiger M, Thome UH, Maier RF, et al. Effects of liberal vs restrictive transfusion thresholds on survival and neurocognitive outcomes in extremely low-birth-weight infants: the ETTNO randomized clinical trial. JAMA. 2020;324(6):560–70. 32780138 10.1001/jama.2020.10690 PMC7420159

[3] Juul SE, Comstock BA, Wadhawan R, Mayock DE, Courtney SE, Robinson T, et al. A randomized trial of erythropoietin for neuroprotection in preterm infants. N Engl J Med. 2020;382(3):233–43. 31940698 10.1056/NEJMoa1907423 PMC7060076

[4] Hansen ML, Pellicer A, Hyttel-Sorensen S, Ergenekon E, Szczapa T, Hagmann C, et al. Cerebral oximetry monitoring in extremely preterm infants. N Engl J Med. 2023;388(16):1501–11. 37075142 10.1056/NEJMoa2207554

[5] Kirpalani H, Bell EF, Hintz SR, Tan S, Schmidt B, Chaudhary AS, et al. Higher or lower hemoglobin transfusion thresholds for preterm infants. N Engl J Med. 2020;383(27):2639–51. 33382931 10.1056/NEJMoa2020248 PMC8487591

[6] Carlo WA, Goudar SS, Jehan I, Chomba E, Tshefu A, Garces A, et al. Newborn-care training and perinatal mortality in developing countries. N Engl J Med. 2010;362(7):614–23. 20164485 10.1056/NEJMsa0806033 PMC3565382

[7] Group BIUKC, Group BIAC, Group BINZC, Stenson BJ, Tarnow-Mordi WO, Darlow BA, Group BIUKC, Group BIAC, Group BINZC, et al. Oxygen saturation and outcomes in preterm infants. N Engl J Med. 2013;368(22):2094–104. 23642047 10.1056/NEJMoa1302298

[8] Darlow BA, Marschner SL, Donoghoe M, Battin MR, Broadbent RS, Elder MJ, et al. Randomized controlled trial of oxygen saturation targets in very preterm infants: two year outcomes. J Pediatr. 2014;165(1):30-5 e2. 24560181 10.1016/j.jpeds.2014.01.017

[9] Saugstad OD, Aune D. Optimal oxygenation of extremely low birth weight infants: a meta-analysis and systematic review of the oxygen saturation target studies. Neonatology. 2014;105(1):55–63. 24247112 10.1159/000356561

[10] Di Fiore JM, Bloom JN, Orge F, Schutt A, Schluchter M, Cheruvu VK, et al. A higher incidence of intermittent hypoxemic episodes is associated with severe retinopathy of prematurity. J Pediatr. 2010;157(1):69–73. 20304417 10.1016/j.jpeds.2010.01.046 PMC4428609

[11] Cunningham S, Fleck BW, Elton RA, McIntosh N. Transcutaneous oxygen levels in retinopathy of prematurity. Lancet. 1995;346(8988):1464–5. 7490994 10.1016/s0140-6736(95)92475-2

[12] Di Fiore JM, Kaffashi F, Loparo K, Sattar A, Schluchter M, Foglyano R, et al. The relationship between patterns of intermittent hypoxia and retinopathy of prematurity in preterm infants. Pediatr Res. 2012;72(6):606–12. 23037873 10.1038/pr.2012.132 PMC4433009

[13] Poets CF, Roberts RS, Schmidt B, Whyte RK, Asztalos EV, Bader D, et al. Association between intermittent hypoxemia or bradycardia and late death or disability in extremely preterm infants. JAMA. 2015;314(6):595–603. 26262797 10.1001/jama.2015.8841

[14] Martin RJ, Wang K, Koroglu O, Di Fiore J, Kc P. Intermittent hypoxic episodes in preterm infants: do they matter? Neonatology. 2011;100(3):303–10. 21986336 10.1159/000329922 PMC3252018

[15] Lim K, Wheeler KI, Gale TJ, Jackson HD, Kihlstrand JF, Sand C, et al. Oxygen saturation targeting in preterm infants receiving continuous positive airway pressure. J Pediatr. 2014;164(4):730-6 e1. 24433828 10.1016/j.jpeds.2013.11.072

[16] Claure N, Bancalari E, D’Ugard C, Nelin L, Stein M, Ramanathan R, et al. Multicenter crossover study of automated control of inspired oxygen in ventilated preterm infants. Pediatrics. 2011;127(1):e76-83. 21187305 10.1542/peds.2010-0939

[17] Hallenberger A, Poets CF, Horn W, Seyfang A, Urschitz MS, Group CS. Closed-loop automatic oxygen control (CLAC) in preterm infants: a randomized controlled trial. Pediatrics. 2014;133(2):e379–85. 24470641 10.1542/peds.2013-1834

[18] Schouten TMR, Abu-Hanna A, van Kaam AH, van den Heuvel MEN, Bachman TE, van Leuteren RW, et al. Prolonged use of closed-loop inspired oxygen support in preterm infants: a randomised controlled trial. Arch Dis Child Fetal Neonatal Ed. 2024;109(2):221–6. 37827816 10.1136/archdischild-2023-325831

[19] Schwarz CE, Kreutzer KB, Langanky L, Wolf NS, Braun W, O’Sullivan MP, et al. Randomised crossover trial comparing algorithms and averaging times for automatic oxygen control in preterm infants. Arch Dis Child Fetal Neonatal Ed. 2022;107(4):425–30. 34819347 10.1136/archdischild-2021-322096

[20] Dijkman KP, Mohns T, Dieleman JP, van Pul C, Goos TG, Reiss IK, et al. Predictive Intelligent Control of Oxygenation (PRICO) in preterm infants on high flow nasal cannula support: a randomised cross-over study. Arch Dis Child Fetal Neonatal Ed. 2021;106(6):621–6. 33972265 10.1136/archdischild-2020-320728

[21] Nair V, Kannan Loganathan P, Lal MK, Pringleton H, Bachman TE, Brodlie M, et al. Automatic oxygen control for reducing extremes of oxygen saturation: a randomised controlled trial. Arch Dis Child Fetal Neonatal Ed. 2023;108(2):136–41. 35999043 10.1136/archdischild-2022-324160

[22] Sturrock S, Ambulkar H, Williams EE, Sweeney S, Bednarczuk NF, Dassios T, et al. A randomised crossover trial of closed loop automated oxygen control in preterm, ventilated infants. Acta Paediatr. 2021;110(3):833–7. 32969040 10.1111/apa.15585

[23] Schwarz CE, Kidszun A, Bieder NS, Franz AR, Konig J, Mildenberger E, et al. Is faster better? A randomised crossover study comparing algorithms for closed-loop automatic oxygen control. Arch Dis Child Fetal Neonatal Ed. 2020;105(4):369–74. 31527093 10.1136/archdischild-2019-317029

[24] Gajdos M, Waitz M, Mendler MR, Braun W, Hummler H. Effects of a new device for automated closed loop control of inspired oxygen concentration on fluctuations of arterial and different regional organ tissue oxygen saturations in preterm infants. Arch Dis Child Fetal Neonatal Ed. 2019;104(4):F360–5. 30154236 10.1136/archdischild-2018-314769

[25] Reynolds PR, Miller TL, Volakis LI, Holland N, Dungan GC, Roehr CC, et al. Randomised cross-over study of automated oxygen control for preterm infants receiving nasal high flow. Arch Dis Child Fetal Neonatal Ed. 2019;104(4):F366–71. 30464005 10.1136/archdischild-2018-315342

[26] Van Zanten HA, Kuypers K, Stenson BJ, Bachman TE, Pauws SC, Te Pas AB. The effect of implementing an automated oxygen control on oxygen saturation in preterm infants. Arch Dis Child Fetal Neonatal Ed. 2017;102(5):F395–9. 28209638 10.1136/archdischild-2016-312172

[27] Salverda HH, Cramer SJE, Witlox R, Dargaville PA, Te Pas AB. Automated oxygen control in preterm infants, how does it work and what to expect: a narrative review. Arch Dis Child Fetal Neonatal Ed. 2021;106(2):215–21. 32732378 10.1136/archdischild-2020-318918

[28] Mitra S, McMillan D. Automated control of fraction of inspired oxygen: is it time for widespread adoption? Curr Opin Pediatr. 2021;33(2):209–16. 33394746 10.1097/MOP.0000000000000993

[29] Poets CF, Franz AR. Automated FiO2 control: nice to have, or an essential addition to neonatal intensive care? Arch Dis Child Fetal Neonatal Ed. 2017;102(1):F5–6. 27698194 10.1136/archdischild-2016-311647

[30] Claure N, Bancalari E. Closed-loop control of inspired oxygen in premature infants. Semin Fetal Neonatal Med. 2015;20(3):198–204. 25773271 10.1016/j.siny.2015.02.003

[31] Dargaville PA, Marshall AP, McLeod L, Salverda HH, Te Pas AB, Gale TJ. Automation of oxygen titration in preterm infants: current evidence and future challenges. Early Hum Dev. 2021;162:105462. 34511288 10.1016/j.earlhumdev.2021.105462

[32] Mitra S, Singh B, El-Naggar W, McMillan DD. Automated versus manual control of inspired oxygen to target oxygen saturation in preterm infants: a systematic review and meta-analysis. J Perinatol. 2018;38(4):351–60. 29296004 10.1038/s41372-017-0037-z

[33] Schulz KF, Altman DG, Moher D, Group C. CONSORT 2010 statement: updated guidelines for reporting parallel group randomised trials. BMJ. 2010;340:c332. 20332509 10.1136/bmj.c332 PMC2844940

[34] Palisano R, Rosenbaum P, Walter S, Russell D, Wood E, Galuppi B. Development and reliability of a system to classify gross motor function in children with cerebral palsy. Dev Med Child Neurol. 1997;39(4):214–23. 9183258 10.1111/j.1469-8749.1997.tb07414.x

[35] International Committee for the Classification of Retinopathy of P. The international classification of retinopathy of prematurity revisited. Arch Ophthalmol. 2005;123(7):991–9. 16009843 10.1001/archopht.123.7.991

[36] Walsh MC, Wilson-Costello D, Zadell A, Newman N, Fanaroff A. Safety, reliability, and validity of a physiologic definition of bronchopulmonary dysplasia. J Perinatol. 2003;23(6):451–6. 13679930 10.1038/sj.jp.7210963

[37] Bell MJ, Ternberg JL, Feigin RD, Keating JP, Marshall R, Barton L, et al. Neonatal necrotizing enterocolitis. Therapeutic decisions based upon clinical staging. Ann Surg. 1978;187(1):1–7. 413500 10.1097/00000658-197801000-00001 PMC1396409

[38] Smith LEH, Hellström A, Stahl A, Fielder A, Chambers W, Moseley J, et al. Development of a retinopathy of prematurity activity scale and clinical outcome measures for use in clinical trials. Jama Ophthalmol. 2019;137(3):305–11. 30543348 10.1001/jamaophthalmol.2018.5984 PMC6565513

[39] van Elteren PH. On the combination of independent two-sample tests of Wilcoxon. Bulletin of the International Statistical Institute. 1960;37:351–61.

[40] Bender R, Vervolgyi V. Estimating adjusted NNTs in randomised controlled trials with binary outcomes: a simulation study. Contemp Clin Trials. 2010;31(5):498–505. 20624484 10.1016/j.cct.2010.07.005

[41] Bishop YMM, Fienberg SE, Holland PW. Discrete multivariate analysis: theory and practice. Cambridge, Mass: MIT Press; 1975.

[42] L’Abbe KA, Detsky AS, O’Rourke K. Meta-analysis in clinical research. Ann Intern Med. 1987;107(2):224–33. 3300460 10.7326/0003-4819-107-2-224

[43] Schwarz CE, Lightbody G, Muller-Hansen I, Arand J, Poets CF, Franz AR. In vitro evaluation of delays in the adjustment of the fraction of inspired oxygen during CPAP: effect of flow and volume. Arch Dis Child Fetal Neonatal Ed. 2021;106(2):205–7. 32796056 10.1136/archdischild-2020-319058

[44] Maiwald CA, Schwarz CE, Bockmann K, Springer L, Poets CF, Franz A. Randomised crossover study on pulse oximeter readings from different sensors in very preterm infants. Arch Dis Child Fetal Neonatal Ed. 2024;109(4):391–6. 38129130 10.1136/archdischild-2023-325961 PMC11228211

